# *N*-Butyl-4-butyl­imino-2-methyl­pentan-2-aminium (*E*)-quercetinate

**DOI:** 10.1107/S1600536812031170

**Published:** 2012-07-14

**Authors:** Ioana-Georgeta Grosu, Gheorghe Borodi, Mihaela Maria Pop

**Affiliations:** a National Institute for R&D of Isotopic and Molecular Technologies, PO Box 700, Cluj-Napoca R-400293, Romania

## Abstract

The title salt, C_14_H_31_N_2_^+^·C_15_H_9_O_7_^−^, was obtained in the reaction of quercetin with *n*-butyl­amine in a mixture of acetone and hexane. The crystal structure determination shows that the quercetin donates one of its phenol H atoms to the *N*-butyl-4-butyl­imino-2-methyl­pentan-2-amine mol­ecule. The crystal structure of the salt is stabilized by intramolecular (N—H⋯N for the cation and O—H⋯O for the anion) and intermolecular hydrogen bonding (N—H⋯O between cation–anion pairs and O—H⋯O between anions). Quercetin molecules form dimers connected into a two-dimensional network. The dihedral angle between the quercetin ring systems is 19.61 (8)°.

## Related literature

For the anti­oxidant activity of quercetin, see: Young *et al.* (1999 ▶). For related co-crystal structures, see: Clarke *et al.* (2010 ▶); Kavuru *et al.* (2010 ▶).
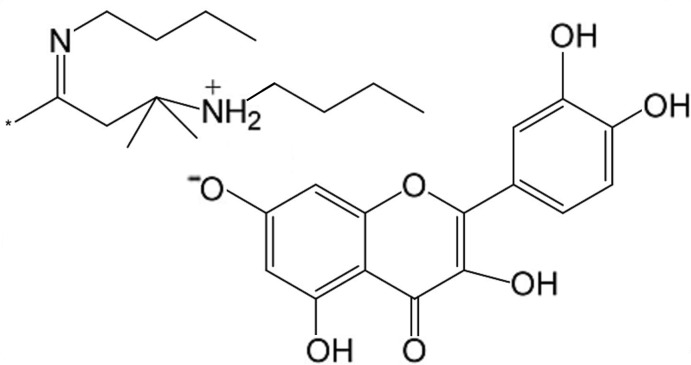


## Experimental

### 

#### Crystal data

C_14_H_31_N_2_^+^·C_15_H_9_O_7_^−^
*M* *_r_* = 528.63
Monoclinic, 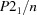

*a* = 11.4017 (7) Å
*b* = 13.1730 (5) Å
*c* = 19.1961 (9) Å
β = 104.438 (6)°
*V* = 2792.1 (2) Å^3^
*Z* = 4
Mo *K*α radiation
μ = 0.09 mm^−1^
*T* = 293 K
0.3 × 0.2 × 0.1 mm

#### Data collection

SuperNova, Dual, Cu at zero, Eos diffractometer
Absorption correction: multi-scan (*CrysAlis PRO*; Oxford Diffraction, 2010 ▶). *T*_min_ = 0.647, *T*_max_ = 1.000
25155 measured reflections
6574 independent reflections
4881 reflections with *I* > 2σ(*I*)
*R*_int_ = 0.030

#### Refinement

*R*[*F* ^2^ > 2σ(*F* ^2^)] = 0.065
*wR*(*F* ^2^) = 0.233
*S* = 1.57
6574 reflections
352 parameters
H-atom parameters constrained
Δρ_max_ = 0.41 e Å^−3^
Δρ_min_ = −0.38 e Å^−3^

### 

Data collection: *CrysAlis PRO* (Oxford Diffraction, 2010 ▶); cell refinement: *CrysAlis PRO*; data reduction: *CrysAlis PRO*; program(s) used to solve structure: *SHELXS97* (Sheldrick, 2008 ▶); program(s) used to refine structure: *SHELXL97* (Sheldrick, 2008 ▶); molecular graphics: *Mercury* (Macrae *et al.*, 2006 ▶); software used to prepare material for publication: *OLEX2* (Dolomanov *et al.*, 2009 ▶).

## Supplementary Material

Crystal structure: contains datablock(s) global, I. DOI: 10.1107/S1600536812031170/kj2204sup1.cif

Structure factors: contains datablock(s) I. DOI: 10.1107/S1600536812031170/kj2204Isup2.hkl

Supplementary material file. DOI: 10.1107/S1600536812031170/kj2204Isup3.cml

Additional supplementary materials:  crystallographic information; 3D view; checkCIF report

## Figures and Tables

**Table 1:** Hydrogen-bond geometry (Å, °)

*D*—H⋯*A*	*D*—H	H⋯*A*	*D*⋯*A*	*D*—H⋯*A*
N2*B*—H2*BA*⋯O6*A*	0.9	1.87	2.765 (2)	171
N2*B*—H2*BB*⋯N1*B*	0.9	2.05	2.749 (3)	134
O7*A*—H7*A*⋯O5*A*	0.82	1.92	2.642 (2)	147
O1*A*—H1*A*⋯O6*A* ^i^	0.82	1.73	2.544 (2)	172
O2*A*—H2*A*⋯O6*A* ^i^	0.82	1.85	2.6637 (19)	173
O4*A*—H4*A*⋯O2*A* ^ii^	0.82	2.01	2.771 (2)	154

Symmetry codes: (i) 
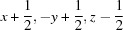
; (ii) 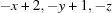.

**Fig. 1.**
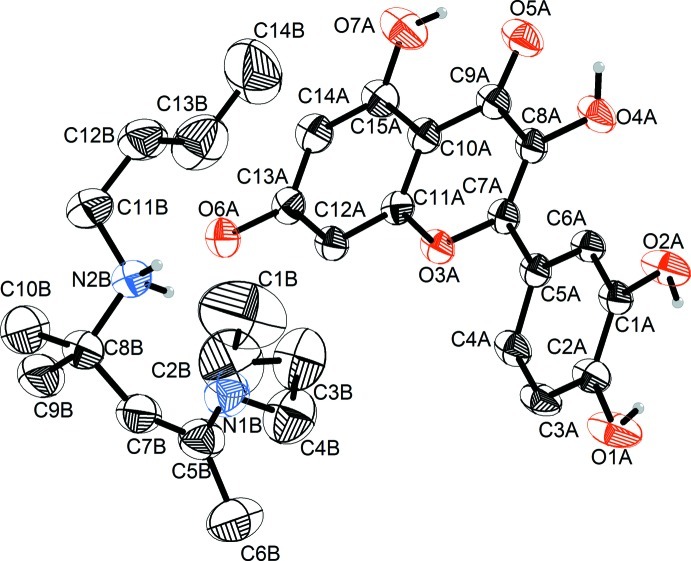
The molecular structure of (I) showing the atomic numbering and 50% probability displacement ellipsoid. The C-bound H atoms are omitted for clarity.

**Fig. 2.**
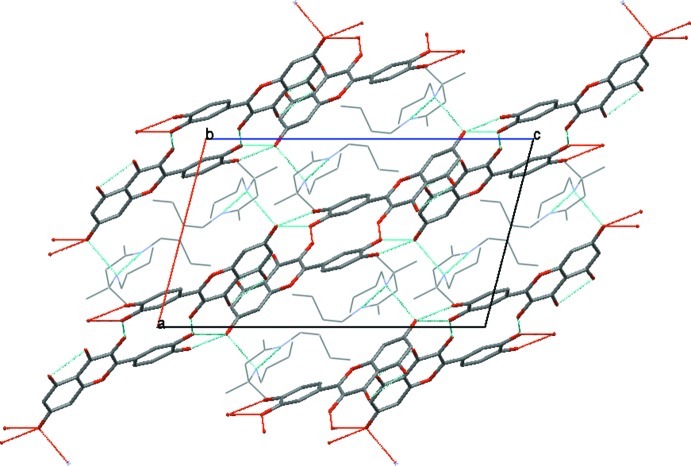
A view along the b axis of the crystal packing of the title compound. The hydrogen bonds are shown as dashed lines and the H atoms have been omitted for clarity.

## supplementary crystallographic information

### Comment

Quercetin belongs to the class of flavonoids which are naturally existing polyphenols possessing anti-oxidant activity (Young *et al., 1999*). Co-crystal forms of quercetin with theobromine and isonicotinic acid were reported (Clarke *et al.*, 2010 and Kavuru *et al.*, 2010). We present here the crystal structure of the title compound (Fig. 1). Quercetin molecules form nearly planar dimers through hydroxyl-hydroxyl (O4A—H4*A*···O2*A*^ii^, Table 1) supra-molecular homosynthon. The dihedral angle between the quercetin ring systems is 19.61 (8) degrees. The quercetin dimers are further connected *via* OH···O intermolecular hydrogen bonding involving the phenyl moieties into an infinite two-dimensional network (base vectors [1 0 - 1], [0 - 1 0]), extending parallel to (1 0 1) as shown in Fig. 2. One phenolic OH of quercetin is engaged in hydrogen bonding with one molecule of *N*-butyl-4-(butylimino)-2-methylpentane-2-amine and it transfers the proton to the basic moiety. Therefore, the title compound is a salt and not a co-crystal as the ones reported for quercetin so far.

### Experimental

The title compound (C_15_H_9_O_7_) (C_14_H_31_N_2_) was obtained in the reaction of quercetin with n-butylamine in a mixture of acetone/hexane. A suspension of quercetin dihydrate (0.044 mmol) in a mixture of acetone (2 ml) and hexane (1 ml) was stirred at 333 K for 30 minutes. The suspension was filtered and the clear solution was placed in a vial. The vial containing the quercetin solution was placed in a larger vial containing n-butylamine (2.5 ml). The vial was sealed to allow the slow diffusion of the amine vapors into the acetone/hexane quercetin solution. Yellow crystals of (I) were obtained after three days.

### Refinement

All H atoms were located in a difference map. The hydrogen atoms of the methyl and hydroxyl groups were allowed to rotate to best fit the experimental electron density, whilst keeping fixed angles and distances (d(C-H) = 0.96 Å, d(O-H) = 0.82 Å), with U(H) set to 1.5 U_eq_ (C,O). The remaining H atoms were placed in the calculated positions with d(C-H) = 0.97 Å (CH2 groups), d(C-H) = 0.93 Å (aromatic ring) and d(N-H) = 0.9 Å. They were included in the refinement in the riding model approximation, with U(H) set to 1.2 U_eq_ (C,N).

### Crystal data

**Table:**

C_14_H_31_N_2_^+^·C_15_H_9_O_7_^−^	*F*(000) = 1136
*M**_r_* = 528.63	*D*_x_ = 1.258 Mg m^−^^3^
Monoclinic, *P*2_1_/*n*	Mo *K*α radiation, λ = 0.7107 Å
*a* = 11.4017 (7) Å	Cell parameters from 9486 reflections
*b* = 13.1730 (5) Å	θ = 3.1–28.9°
*c* = 19.1961 (9) Å	µ = 0.09 mm^−^^1^
β = 104.438 (6)°	*T* = 293 K
*V* = 2792.1 (2) Å^3^	Prism, yellow
*Z* = 4	0.3 × 0.2 × 0.1 mm

### Data collection

**Table:**

SuperNova, Dual, Cu at zero, Eos diffractometer	6574 independent reflections
Radiation source: SuperNova (Mo) X-ray Source	4881 reflections with *I* > 2σ(*I*)
Mirror monochromator	*R*_int_ = 0.030
Detector resolution: 16.4335 pixels mm^-1^	θ_max_ = 29.0°, θ_min_ = 3.1°
ω scans	*h* = −14→15
Absorption correction: multi-scan (*CrysAlis PRO*; Oxford Diffraction, 2010).	*k* = −16→17
*T*_min_ = 0.647, *T*_max_ = 1.000	*l* = −24→26
25155 measured reflections	

### Refinement

**Table:**

Refinement on *F*^2^	Primary atom site location: structure-invariant direct methods
Least-squares matrix: full	Secondary atom site location: difference Fourier map
*R*[*F*^2^ > 2σ(*F*^2^)] = 0.065	Hydrogen site location: inferred from neighbouring sites
*wR*(*F*^2^) = 0.233	H-atom parameters constrained
*S* = 1.57	*w* = 1/[σ^2^(*F*_o_^2^) + (0.1*P*)^2^] where *P* = (*F*_o_^2^ + 2*F*_c_^2^)/3
6574 reflections	(Δ/σ)_max_ < 0.001
352 parameters	Δρ_max_ = 0.41 e Å^−^^3^
0 restraints	Δρ_min_ = −0.38 e Å^−^^3^

### Special details

**Table:**

Geometry. All e.s.d.'s (except the e.s.d. in the dihedral angle between two l.s. planes) are estimated using the full covariance matrix. The cell e.s.d.'s are taken into account individually in the estimation of e.s.d.'s in distances, angles and torsion angles; correlations between e.s.d.'s in cell parameters are only used when they are defined by crystal symmetry. An approximate (isotropic) treatment of cell e.s.d.'s is used for estimating e.s.d.'s involving l.s. planes.
Refinement. Refinement of *F*^2^ against ALL reflections. The weighted *R*-factor *wR* and goodness of fit *S* are based on *F*^2^, conventional *R*-factors *R* are based on *F*, with *F* set to zero for negative *F*^2^. The threshold expression of *F*^2^ > σ(*F*^2^) is used only for calculating *R*-factors(gt) *etc*. and is not relevant to the choice of reflections for refinement. *R*-factors based on *F*^2^ are statistically about twice as large as those based on *F*, and *R*- factors based on ALL data will be even larger.

### Fractional atomic coordinates and isotropic or equivalent isotropic displacement parameters (Å^2^)

**Table:**

	*x*	*y*	*z*	*U*_iso_*/*U*_eq_	
O3A	0.72169 (12)	0.32311 (9)	0.12724 (7)	0.0363 (3)	
O6A	0.46443 (12)	0.32713 (10)	0.28345 (7)	0.0406 (3)	
C12A	0.59541 (17)	0.32353 (14)	0.20600 (9)	0.0348 (4)	
H12A	0.5636	0.2617	0.1867	0.042*	
O4A	0.94607 (14)	0.49703 (10)	0.09262 (8)	0.0487 (4)	
H4A	0.9538	0.5572	0.1038	0.073*	
C7A	0.80878 (16)	0.36415 (13)	0.09754 (9)	0.0331 (4)	
C5A	0.83343 (17)	0.29650 (12)	0.04206 (9)	0.0325 (4)	
O5A	0.87994 (14)	0.59111 (10)	0.20140 (8)	0.0507 (4)	
O2A	0.96400 (16)	0.30683 (10)	−0.11267 (8)	0.0557 (5)	
H2A	0.9692	0.2635	−0.1424	0.084*	
C10A	0.73580 (17)	0.46380 (13)	0.20802 (9)	0.0351 (4)	
O7A	0.73998 (17)	0.59868 (12)	0.29219 (9)	0.0643 (5)	
H7A	0.7868	0.6201	0.2694	0.097*	
C11A	0.68442 (16)	0.37099 (13)	0.18113 (9)	0.0321 (4)	
C9A	0.82744 (17)	0.50911 (13)	0.17907 (10)	0.0365 (4)	
C14A	0.60419 (19)	0.46257 (15)	0.28970 (10)	0.0423 (5)	
H14A	0.5774	0.4929	0.3267	0.051*	
C8A	0.85987 (17)	0.45473 (13)	0.12110 (10)	0.0348 (4)	
C6A	0.89019 (16)	0.33040 (13)	−0.01056 (9)	0.0332 (4)	
H6A	0.9152	0.3977	−0.0100	0.040*	
C13A	0.55355 (17)	0.36998 (14)	0.26088 (9)	0.0352 (4)	
C1A	0.90966 (17)	0.26601 (13)	−0.06322 (9)	0.0347 (4)	
C2A	0.87269 (19)	0.16429 (14)	−0.06480 (10)	0.0407 (5)	
C4A	0.79879 (19)	0.19478 (14)	0.04075 (11)	0.0411 (5)	
H4AA	0.7619	0.1701	0.0754	0.049*	
C15A	0.69287 (19)	0.50915 (15)	0.26400 (10)	0.0421 (5)	
C3A	0.8190 (2)	0.13110 (14)	−0.01161 (11)	0.0454 (5)	
H3A	0.7960	0.0634	−0.0114	0.054*	
N2B	0.27813 (17)	0.27896 (12)	0.16548 (11)	0.0500 (5)	
H2BA	0.3331	0.2946	0.2065	0.060*	
H2BB	0.3193	0.2632	0.1326	0.060*	
N1B	0.39679 (19)	0.13238 (17)	0.10738 (12)	0.0644 (6)	
C8B	0.2110 (2)	0.18603 (17)	0.17890 (15)	0.0600 (6)	
C12B	0.2816 (3)	0.46167 (19)	0.13476 (16)	0.0687 (7)	
H12B	0.2315	0.5220	0.1301	0.082*	
H12C	0.3435	0.4675	0.1796	0.082*	
C7B	0.3047 (2)	0.10201 (17)	0.20315 (14)	0.0590 (6)	
H7BA	0.3593	0.1235	0.2480	0.071*	
H7BB	0.2624	0.0423	0.2137	0.071*	
C5B	0.3785 (2)	0.07113 (18)	0.15472 (14)	0.0602 (6)	
C3B	0.4607 (3)	0.1825 (3)	−0.00001 (18)	0.0922 (10)	
H3BA	0.5218	0.1684	−0.0260	0.111*	
H3BB	0.4758	0.2498	0.0207	0.111*	
C9B	0.1187 (2)	0.1555 (2)	0.11018 (18)	0.0795 (9)	
H9BA	0.0567	0.2065	0.0981	0.119*	
H9BB	0.0829	0.0917	0.1174	0.119*	
H9BC	0.1583	0.1492	0.0717	0.119*	
C13B	0.3421 (3)	0.4609 (3)	0.0749 (2)	0.0939 (10)	
H13A	0.2810	0.4594	0.0295	0.113*	
H13B	0.3906	0.3998	0.0779	0.113*	
C6B	0.4337 (3)	−0.0340 (2)	0.16675 (19)	0.0844 (9)	
H6BA	0.3785	−0.0825	0.1389	0.127*	
H6BB	0.4492	−0.0512	0.2168	0.127*	
H6BC	0.5083	−0.0351	0.1522	0.127*	
C11B	0.2045 (3)	0.37186 (18)	0.14017 (18)	0.0738 (8)	
H11A	0.1504	0.3585	0.0934	0.089*	
H11B	0.1551	0.3872	0.1733	0.089*	
C10B	0.1493 (3)	0.2091 (2)	0.2401 (2)	0.0884 (10)	
H10A	0.2089	0.2323	0.2817	0.133*	
H10B	0.1115	0.1486	0.2519	0.133*	
H10C	0.0891	0.2609	0.2247	0.133*	
C4B	0.4711 (3)	0.1058 (3)	0.05941 (18)	0.0874 (9)	
H4BA	0.4468	0.0396	0.0385	0.105*	
H4BB	0.5549	0.1012	0.0866	0.105*	
C14B	0.4228 (4)	0.5531 (3)	0.0763 (3)	0.1369 (19)	
H14B	0.3809	0.6130	0.0854	0.205*	
H14C	0.4425	0.5596	0.0307	0.205*	
H14D	0.4958	0.5450	0.1136	0.205*	
C1B	0.3270 (6)	0.2454 (5)	−0.1144 (3)	0.175 (3)	
H1BA	0.3371	0.3149	−0.0990	0.263*	
H1BB	0.2487	0.2368	−0.1469	0.263*	
H1BC	0.3887	0.2276	−0.1383	0.263*	
C2B	0.3368 (4)	0.1799 (4)	−0.0518 (3)	0.1251 (14)	
H2BC	0.3185	0.1106	−0.0682	0.150*	
H2BD	0.2769	0.2007	−0.0266	0.150*	
O1A	0.88657 (18)	0.09668 (11)	−0.11484 (9)	0.0658 (5)	
H1A	0.9087	0.1263	−0.1469	0.099*	

### Atomic displacement parameters (Å^2^)

**Table:**

	*U*^11^	*U*^22^	*U*^33^	*U*^12^	*U*^13^	*U*^23^
O3A	0.0456 (8)	0.0369 (7)	0.0352 (7)	−0.0082 (5)	0.0265 (6)	−0.0079 (5)
O6A	0.0461 (8)	0.0475 (7)	0.0370 (7)	−0.0029 (6)	0.0267 (6)	0.0032 (5)
C12A	0.0403 (11)	0.0365 (9)	0.0325 (9)	−0.0025 (7)	0.0181 (8)	−0.0008 (7)
O4A	0.0599 (10)	0.0430 (8)	0.0573 (9)	−0.0183 (6)	0.0415 (8)	−0.0152 (6)
C7A	0.0378 (10)	0.0378 (9)	0.0294 (8)	−0.0020 (7)	0.0192 (7)	−0.0010 (7)
C5A	0.0363 (10)	0.0352 (9)	0.0305 (8)	−0.0023 (7)	0.0171 (7)	−0.0029 (7)
O5A	0.0632 (10)	0.0441 (8)	0.0543 (9)	−0.0195 (7)	0.0325 (7)	−0.0165 (6)
O2A	0.0947 (12)	0.0401 (7)	0.0511 (9)	−0.0205 (7)	0.0534 (9)	−0.0132 (6)
C10A	0.0397 (11)	0.0392 (9)	0.0309 (9)	−0.0024 (7)	0.0172 (7)	−0.0040 (7)
O7A	0.0866 (13)	0.0574 (9)	0.0655 (10)	−0.0290 (8)	0.0499 (9)	−0.0337 (8)
C11A	0.0389 (10)	0.0346 (9)	0.0272 (8)	0.0015 (7)	0.0163 (7)	−0.0011 (6)
C9A	0.0418 (11)	0.0376 (9)	0.0339 (9)	−0.0057 (7)	0.0167 (8)	−0.0051 (7)
C14A	0.0524 (13)	0.0477 (10)	0.0352 (9)	−0.0020 (9)	0.0265 (9)	−0.0090 (8)
C8A	0.0381 (10)	0.0372 (9)	0.0350 (9)	−0.0030 (7)	0.0204 (8)	−0.0028 (7)
C6A	0.0420 (11)	0.0308 (8)	0.0319 (9)	−0.0040 (7)	0.0184 (7)	−0.0021 (7)
C13A	0.0403 (11)	0.0421 (9)	0.0286 (8)	0.0024 (8)	0.0186 (7)	0.0049 (7)
C1A	0.0433 (11)	0.0345 (8)	0.0329 (9)	−0.0043 (7)	0.0220 (8)	−0.0007 (7)
C2A	0.0545 (13)	0.0367 (9)	0.0389 (10)	−0.0092 (8)	0.0265 (9)	−0.0097 (7)
C4A	0.0515 (12)	0.0404 (9)	0.0404 (10)	−0.0108 (8)	0.0283 (9)	−0.0051 (8)
C15A	0.0517 (13)	0.0438 (10)	0.0369 (9)	−0.0076 (8)	0.0223 (9)	−0.0103 (8)
C3A	0.0644 (14)	0.0340 (9)	0.0480 (11)	−0.0142 (8)	0.0335 (10)	−0.0080 (8)
N2B	0.0492 (11)	0.0421 (9)	0.0576 (11)	0.0029 (7)	0.0115 (8)	0.0013 (8)
N1B	0.0646 (14)	0.0664 (13)	0.0640 (13)	0.0019 (10)	0.0192 (11)	−0.0097 (11)
C8B	0.0535 (15)	0.0461 (12)	0.0791 (17)	−0.0036 (10)	0.0140 (12)	0.0046 (11)
C12B	0.0732 (18)	0.0513 (13)	0.0774 (18)	0.0080 (12)	0.0106 (14)	0.0126 (12)
C7B	0.0611 (16)	0.0458 (12)	0.0670 (15)	−0.0023 (10)	0.0103 (12)	0.0027 (10)
C5B	0.0614 (16)	0.0512 (13)	0.0614 (15)	−0.0032 (10)	0.0031 (12)	−0.0038 (11)
C3B	0.088 (2)	0.121 (3)	0.0681 (19)	0.0099 (19)	0.0205 (17)	−0.0051 (18)
C9B	0.0555 (17)	0.0577 (14)	0.111 (2)	−0.0062 (12)	−0.0055 (15)	−0.0007 (15)
C13B	0.103 (2)	0.093 (2)	0.089 (2)	0.0237 (19)	0.0301 (19)	0.0281 (18)
C6B	0.093 (2)	0.0599 (16)	0.093 (2)	0.0146 (15)	0.0105 (17)	−0.0055 (15)
C11B	0.0647 (17)	0.0482 (13)	0.104 (2)	0.0104 (11)	0.0132 (15)	0.0108 (13)
C10B	0.093 (2)	0.0713 (18)	0.118 (3)	0.0025 (16)	0.059 (2)	0.0081 (18)
C4B	0.093 (2)	0.097 (2)	0.0758 (19)	0.0072 (18)	0.0288 (17)	−0.0140 (17)
C14B	0.108 (3)	0.126 (3)	0.183 (5)	−0.001 (2)	0.046 (3)	0.079 (3)
C1B	0.190 (6)	0.140 (4)	0.143 (5)	−0.015 (4)	−0.059 (4)	0.032 (4)
C2B	0.123 (3)	0.143 (4)	0.101 (3)	−0.001 (3)	0.014 (3)	0.024 (3)
O1A	0.1151 (15)	0.0414 (8)	0.0631 (10)	−0.0239 (8)	0.0637 (11)	−0.0208 (7)

### Geometric parameters (Å, º)

**Table:**

O3A—C7A	1.373 (2)	C8B—C10B	1.542 (4)
O3A—C11A	1.3667 (19)	C12B—H12B	0.9700
O6A—C13A	1.326 (2)	C12B—H12C	0.9700
C12A—H12A	0.9300	C12B—C13B	1.481 (4)
C12A—C11A	1.375 (2)	C12B—C11B	1.493 (4)
C12A—C13A	1.401 (2)	C7B—H7BA	0.9700
O4A—H4A	0.8200	C7B—H7BB	0.9700
O4A—C8A	1.358 (2)	C7B—C5B	1.458 (4)
C7A—C5A	1.468 (2)	C5B—C6B	1.515 (4)
C7A—C8A	1.355 (2)	C3B—H3BA	0.9700
C5A—C6A	1.402 (2)	C3B—H3BB	0.9700
C5A—C4A	1.395 (2)	C3B—C4B	1.506 (5)
O5A—C9A	1.257 (2)	C3B—C2B	1.512 (6)
O2A—H2A	0.8200	C9B—H9BA	0.9600
O2A—C1A	1.366 (2)	C9B—H9BB	0.9600
C10A—C11A	1.398 (2)	C9B—H9BC	0.9600
C10A—C9A	1.430 (2)	C13B—H13A	0.9700
C10A—C15A	1.419 (2)	C13B—H13B	0.9700
O7A—H7A	0.8200	C13B—C14B	1.520 (5)
O7A—C15A	1.352 (2)	C6B—H6BA	0.9600
C9A—C8A	1.447 (2)	C6B—H6BB	0.9600
C14A—H14A	0.9300	C6B—H6BC	0.9600
C14A—C13A	1.403 (3)	C11B—H11A	0.9700
C14A—C15A	1.375 (3)	C11B—H11B	0.9700
C6A—H6A	0.9300	C10B—H10A	0.9600
C6A—C1A	1.379 (2)	C10B—H10B	0.9600
C1A—C2A	1.403 (2)	C10B—H10C	0.9600
C2A—C3A	1.385 (2)	C4B—H4BA	0.9700
C2A—O1A	1.348 (2)	C4B—H4BB	0.9700
C4A—H4AA	0.9300	C14B—H14B	0.9600
C4A—C3A	1.372 (3)	C14B—H14C	0.9600
C3A—H3A	0.9300	C14B—H14D	0.9600
N2B—H2BA	0.9000	C1B—H1BA	0.9600
N2B—H2BB	0.9000	C1B—H1BB	0.9600
N2B—C8B	1.500 (3)	C1B—H1BC	0.9600
N2B—C11B	1.495 (3)	C1B—C2B	1.460 (7)
N1B—C5B	1.271 (3)	C2B—H2BC	0.9700
N1B—C4B	1.441 (4)	C2B—H2BD	0.9700
C8B—C7B	1.528 (3)	O1A—H1A	0.8200
C8B—C9B	1.522 (4)		
			
C11A—O3A—C7A	121.74 (13)	C8B—C7B—H7BB	107.7
C11A—C12A—H12A	120.6	H7BA—C7B—H7BB	107.1
C11A—C12A—C13A	118.81 (16)	C5B—C7B—C8B	118.6 (2)
C13A—C12A—H12A	120.6	C5B—C7B—H7BA	107.7
C8A—O4A—H4A	109.5	C5B—C7B—H7BB	107.7
O3A—C7A—C5A	110.56 (14)	N1B—C5B—C7B	120.2 (2)
C8A—C7A—O3A	120.22 (15)	N1B—C5B—C6B	123.6 (3)
C8A—C7A—C5A	129.22 (15)	C7B—C5B—C6B	116.2 (2)
C6A—C5A—C7A	122.38 (15)	H3BA—C3B—H3BB	108.0
C4A—C5A—C7A	119.43 (15)	C4B—C3B—H3BA	109.3
C4A—C5A—C6A	118.19 (15)	C4B—C3B—H3BB	109.3
C1A—O2A—H2A	109.5	C4B—C3B—C2B	111.5 (3)
C11A—C10A—C9A	120.06 (15)	C2B—C3B—H3BA	109.3
C11A—C10A—C15A	117.13 (16)	C2B—C3B—H3BB	109.3
C15A—C10A—C9A	122.81 (16)	C8B—C9B—H9BA	109.5
C15A—O7A—H7A	109.5	C8B—C9B—H9BB	109.5
O3A—C11A—C12A	116.66 (15)	C8B—C9B—H9BC	109.5
O3A—C11A—C10A	120.16 (15)	H9BA—C9B—H9BB	109.5
C12A—C11A—C10A	123.18 (15)	H9BA—C9B—H9BC	109.5
O5A—C9A—C10A	123.90 (16)	H9BB—C9B—H9BC	109.5
O5A—C9A—C8A	119.76 (16)	C12B—C13B—H13A	109.2
C10A—C9A—C8A	116.34 (15)	C12B—C13B—H13B	109.2
C13A—C14A—H14A	119.5	C12B—C13B—C14B	112.2 (4)
C15A—C14A—H14A	119.5	H13A—C13B—H13B	107.9
C15A—C14A—C13A	120.90 (16)	C14B—C13B—H13A	109.2
O4A—C8A—C9A	117.12 (15)	C14B—C13B—H13B	109.2
C7A—C8A—O4A	121.41 (15)	C5B—C6B—H6BA	109.5
C7A—C8A—C9A	121.44 (16)	C5B—C6B—H6BB	109.5
C5A—C6A—H6A	119.3	C5B—C6B—H6BC	109.5
C1A—C6A—C5A	121.33 (15)	H6BA—C6B—H6BB	109.5
C1A—C6A—H6A	119.3	H6BA—C6B—H6BC	109.5
O6A—C13A—C12A	119.50 (17)	H6BB—C6B—H6BC	109.5
O6A—C13A—C14A	120.98 (15)	N2B—C11B—H11A	109.2
C12A—C13A—C14A	119.50 (16)	N2B—C11B—H11B	109.2
O2A—C1A—C6A	116.80 (15)	C12B—C11B—N2B	112.2 (2)
O2A—C1A—C2A	123.14 (15)	C12B—C11B—H11A	109.2
C6A—C1A—C2A	120.06 (15)	C12B—C11B—H11B	109.2
C3A—C2A—C1A	118.10 (16)	H11A—C11B—H11B	107.9
O1A—C2A—C1A	123.96 (16)	C8B—C10B—H10A	109.5
O1A—C2A—C3A	117.94 (16)	C8B—C10B—H10B	109.5
C5A—C4A—H4AA	119.9	C8B—C10B—H10C	109.5
C3A—C4A—C5A	120.12 (16)	H10A—C10B—H10B	109.5
C3A—C4A—H4AA	119.9	H10A—C10B—H10C	109.5
O7A—C15A—C10A	119.52 (17)	H10B—C10B—H10C	109.5
O7A—C15A—C14A	120.01 (16)	N1B—C4B—C3B	111.9 (3)
C14A—C15A—C10A	120.47 (17)	N1B—C4B—H4BA	109.2
C2A—C3A—H3A	118.9	N1B—C4B—H4BB	109.2
C4A—C3A—C2A	122.19 (17)	C3B—C4B—H4BA	109.2
C4A—C3A—H3A	118.9	C3B—C4B—H4BB	109.2
H2BA—N2B—H2BB	107.3	H4BA—C4B—H4BB	107.9
C8B—N2B—H2BA	108.0	C13B—C14B—H14B	109.5
C8B—N2B—H2BB	108.0	C13B—C14B—H14C	109.5
C11B—N2B—H2BA	108.0	C13B—C14B—H14D	109.5
C11B—N2B—H2BB	108.0	H14B—C14B—H14C	109.5
C11B—N2B—C8B	117.04 (19)	H14B—C14B—H14D	109.5
C5B—N1B—C4B	122.0 (2)	H14C—C14B—H14D	109.5
N2B—C8B—C7B	107.19 (19)	H1BA—C1B—H1BB	109.5
N2B—C8B—C9B	109.8 (2)	H1BA—C1B—H1BC	109.5
N2B—C8B—C10B	108.6 (2)	H1BB—C1B—H1BC	109.5
C7B—C8B—C10B	109.1 (2)	C2B—C1B—H1BA	109.5
C9B—C8B—C7B	110.7 (2)	C2B—C1B—H1BB	109.5
C9B—C8B—C10B	111.3 (2)	C2B—C1B—H1BC	109.5
H12B—C12B—H12C	107.3	C3B—C2B—H2BC	108.9
C13B—C12B—H12B	108.2	C3B—C2B—H2BD	108.9
C13B—C12B—H12C	108.2	C1B—C2B—C3B	113.3 (4)
C13B—C12B—C11B	116.4 (3)	C1B—C2B—H2BC	108.9
C11B—C12B—H12B	108.2	C1B—C2B—H2BD	108.9
C11B—C12B—H12C	108.2	H2BC—C2B—H2BD	107.7
C8B—C7B—H7BA	107.7	C2A—O1A—H1A	109.5

### Hydrogen-bond geometry (Å, º)

**Table:**

*D*—H···*A*	*D*—H	H···*A*	*D*···*A*	*D*—H···*A*
N2*B*—H2*BA*···O6*A*	0.9	1.87	2.765 (2)	171
N2*B*—H2*BB*···N1*B*	0.9	2.05	2.749 (3)	134
O7*A*—H7*A*···O5*A*	0.82	1.92	2.642 (2)	147
O1*A*—H1*A*···O6*A*^i^	0.82	1.73	2.544 (2)	172
O2*A*—H2*A*···O6*A*^i^	0.82	1.85	2.6637 (19)	173
O4*A*—H4*A*···O2*A*^ii^	0.82	2.01	2.771 (2)	154

Symmetry codes: (i) *x*+1/2, −*y*+1/2, *z*−1/2; (ii) −*x*+2, −*y*+1, −*z*.
